# Incidence of ventricular arrhythmias in patients with chronic total coronary occlusion: Results of the VACTOR study

**DOI:** 10.1016/j.ijcha.2023.101323

**Published:** 2023-12-18

**Authors:** Amira Assaf, Rafi Sakhi, Roberto Diletti, Alexander Hirsch, Cornelis P. Allaart, Rohit Bhagwandien, Mehran Firouzi, Pieter C. Smits, Mark G. Hoogendijk, Dominic A.M.J. Theuns, Sing-Chien Yap

**Affiliations:** a Department of Cardiology, Erasmus MC, University Medical Center Rotterdam, Rotterdam, The Netherlands; b Department of Radiology and Nuclear Medicine, Erasmus MC, University Medical Center Rotterdam, Rotterdam, The Netherlands; c Department of Cardiology, Amsterdam UMC, Vrije Universiteit Amsterdam, Amsterdam, The Netherlands; d Department of Cardiology, Maasstad Hospital, Rotterdam, The Netherlands

**Keywords:** Chronic total coronary occlusion, Implantable cardioverter-defibrillator, Myocardial infarction, Percutaneous coronary intervention, Sudden cardiac death, Ventricular arrhythmia

## Abstract

**Background:**

A chronic total coronary occlusion (CTO) is associated with ventricular arrhythmias (VA) in patients with an implantable cardioverter-defibrillator (ICD). Limited data is available on the incidence of VA in CTO patients without an ICD.

**Objectives:**

To investigate the incidence of sustained VA in CTO patients after successful CTO revascularization and in patients with untreated CTO or failed CTO revascularization.

**Methods:**

Prospective, multicenter observational pilot study including CTO patients who were not eligible for an ICD and had a left ventricular ejection fraction >35 %. We enrolled patients with a successful CTO revascularization (group A) and patients with untreated CTO or failed CTO revascularization (group B). All patients received an implantable loop recorder with remote monitoring. The primary endpoint was sustained VA.

**Results:**

Ninety patients were enrolled (mean age 63 ± 10 years, 83.3 % man, mean LVEF 55 ± 8 %). Group A (n = 45) had a higher prevalence of CTO in the left anterior descending artery in comparison to group B (n = 45) (28.9 % versus 4.4 %, P = 0.002). Other baseline characteristics were similar. During a median follow-up time of 26 months (IQR, 19–35), five patients (5.6 %) had a sustained VA. There was no difference in the incidence of sustained VA between groups (3-year cumulative event rate: 8.8 % (group A) versus 4.5 % (Group B), log-rank P = 0.71).

**Conclusion:**

Patients with an CTO, who do not qualify for an ICD, have a substantial risk of sustained VA. In our study the incidence was not different between patients with revascularized and those with untreated CTO.

## 1 Introduction

A chronic total coronary occlusion (CTO) is associated with an increased risk of ventricular arrhythmias (VA) in recipients of an implantable cardioverter-defibrillator (ICD), both in a primary and secondary prevention cohorts [1], [2], [3], [4]. This vulnerability for VA may be secondary to residual ischemia in the CTO-related myocardium, despite the development of collaterals [5]. Furthermore, the presence of a scar and ischemic border zone in the CTO territory forms the substrate for reentry VA due to the presence of slow conduction channels and dispersion in repolarization [6], [7].

It seems that a CTO is an important risk modifier in ICD recipients; however, it is uncertain whether CTO patients without severe left ventricular dysfunction (i.e., less extensive scar) will also be at increased risk of VA. This information is important for risk stratification. Furthermore, the antiarrhythmic effect of percutaneous coronary intervention (PCI) of the CTO (CTO-PCI) is unknown. Previous randomized CTO-PCI trials demonstrated positive left ventricular remodeling and improvement of quality of life after CTO revascularization but demonstrated no benefit with regard to all-cause death and cardiovascular death [8], [9]. Interestingly, small paired electrophysiological studies have observed a decrease of the size of the ischemic border zone after successful CTO revascularization suggesting reverse electrical remodeling by restoring coronary blood flow [10], [11].

The aim of the present multicenter study was to describe the incidence of ventricular arrhythmias in CTO patients who are not candidates for an ICD. Two groups were evaluated: 1) patients after successful CTO-PCI; and 2) patients with untreated CTO or failed CTO-PCI. Both groups received continuous arrhythmia monitoring with an implantable loop recorder (ILR).

## 2 Methods

### 2.1 Study design and population

The *Incidence of Ventricular Arrhythmias in patients with Chronic Total Occlusion Recanalization* (VACTOR) study (NCT03475888) was an investigator-initiated, prospective, single-arm, multi-center exploratory pilot study conducted in the Erasmus Medical Center (Rotterdam, The Netherlands), the Amsterdam University Medical Center, location VU Medical Center (Amsterdam, The Netherlands), and the Maasstad Hospital (Rotterdam, The Netherlands).

The study population consisted of patients with recently diagnosed or treated CTO without severe left ventricular (LV) dysfunction (>35 %). Main inclusion criteria were presence of CTO (either successfully percutaneously revascularized within 6 months; failed percutaneous revascularization within 6 months; or untreated) and age ≥18 years. Successful CTO-PCI was defined as final TIMI flow grade ≥2 and a residual stenosis ≤30 % after stent implantation. Main exclusion criteria were eligibility for an ICD according to the 2015 ESC guidelines [12], presence of a cardiac implantable electric device, high risk for infection or active infection, known pregnancy at time of inclusion, and severe co-morbidity. Patients were also excluded if they had severe co-morbidity that may have caused non-compliance with the protocol, confounding of the data or limited life expectancy (i.e., less than one year).

**Fig. 1:**
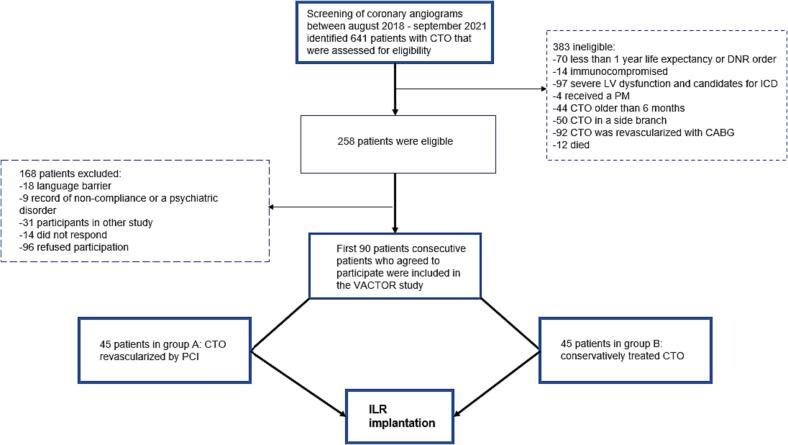
Screening and inclusion of patients in the VACTOR study.

All patients who underwent coronary angiography in the period of September 2018 till October 2021 were screened for the presence of a CTO (Fig. 1). A CTO was defined as complete obstruction of the vessel with Thrombolysis In Myocardial Infarction (TIMI) flow grade 0 and estimated duration of ≥3 months. Eligible patients were included in one of the following two arms: successful CTO-PCI (group A) or untreated CTO or failed CTO-PCI (group B). Both groups received similar medical treatment regimens. The predefined maximum number of patients in each group was 45.

### 2.2 Study endpoints and definitions

The primary endpoint was the occurrence of sustained VA. Sustained VA was defined as VA lasting >30 s or the presence of ventricular fibrillation (VF). Secondary endpoints were the incidence of all-cause mortality, major adverse cardiac events ([MACE], includes either acute myocardial infarction, urgent revascularization, stroke, or death), and actionable arrhythmic events. Actionable arrhythmic events were defined as arrhythmic events leading to a change in clinical management.

Infarct-related artery CTO (IRA-CTO) was defined as a CTO with a myocardial infarction in the territory of the affected coronary artery. Previous myocardial infarction had to be documented by Q waves on ECG and/or evidence of scar on imaging, such as regional wall motion abnormalities on echocardiography or late gadolinium enhancement on cardiac magnetic resonance imaging.

### 2.3 Implantation procedure, device programming and follow-up

For the present study the Medtronic Reveal LINQ ICM system (Medtronic, Minneapolis, MN, USA) was used. Implantation was performed according to the instructions for use of the manufacturer. After implantation, the patient received the MyCareLink Patient Monitor for remote monitoring, as well as instructions about its use for nightly automated transmissions. The ILR was programmed to give the highest priority to VA in the daily wireless audit transmissions. The following settings were used: tachy detection interval 176 bpm, tachy duration 16 beats, brady detection off, pause duration three seconds, atrial tachycardia/atrial fibrillation detection type atrial fibrillation Only, atrial tachycardia/atrial fibrillation recording threshold >10 min. The Reveal LINQ-Patient Assistant was not provided to the patient; thus, symptomatic episodes were not recorded but only automatic detections. After implantation, patients had regular outpatient visits every six months clinic for manual interrogation of the device. The remote monitoring system was checked daily by a technician.

### 2.4 Statistical analysis

Continuous data are presented as mean ± standard deviation or as median with interquartile range, as appropriate. Categorical variables are presented by frequencies and percentages. Differences of continuous variables between groups were analyzed with a Student’s *t*-test or the Mann-Whitney *U* test, depending on normality. Differences between categorical variables were analyzed with the χ^2^ test or Fisher’s exact test, where appropriate. Event rates were estimated with the Kaplan-Meier method, and differences were compared with the log rank test. A p-value < 0.05 was considered statistically significant. Statistical analyses were performed using SPSS V.28 (IBM Corp., New York, USA).

### 2.5 Ethical considerations

This study was conducted in accordance with the Declaration of Helsinki and the international standard for clinical investigation of medical devices in human subjects (ISO 14155). The local ethics committee approved the study (METC-2018-092), and all patients gave written informed consent.

## 3 Results

### 3.1 Study population

A total of 90 patients were enrolled in the study (mean age 63 ± 10 years, 83.3 % male). Most patients had a preserved LV ejection fraction of >50 % (76.7 %); and no or only mild heart failure symptoms (NYHA I-II, 95.6 %). The baseline characteristics between both groups were comparable, except a higher prevalence of a CTO in the left anterior descending artery (LAD) in group A, and a higher prevalence of a CTO in the right coronary artery (RCA) in group B (Table 1).

**Table 1:** Baseline characteristics.

Variable	Total group n = 90	Group A (revascularized CTO) n = 45	Group B (untreated or failed CTO PCI) n = 45	P-value
Age at ILR implantation, years	63 ± 10	62 ± 10	64 ± 10	0.18
Male sex	75 (83.3)	39 (86.7)	36 (80.0)	0.40
Body Mass Index	29 ± 7	30 ± 8	29 ± 6	0.48
Diabetes Mellitus	34 (37.8)	16 (35.6)	18 (40.0)	0.66
Previous CVA or TIA	11 (13.3)	5 (11.1)	6 (13.3)	0.75
Hypertension	52 (57.7)	29 (64.4)	23 (51.1)	0.20
Hypercholesterolemia	44 (48.9)	25 (55.6)	19 (42.2)	0.44
Paroxysmal or persistent AF	15 (16.7)	6 (13.3)	9 (20.2)	0.40
eGFR < 45 ml/min	3 (3.3)	2 (4.4)	1 (2.2)	0.56
Previous MI	45 (50.0)	19 (42.2)	26 (57.8)	0.14
LVEF % LVEF Range	55 ± 8 (36–68)	55 ± 9 (36–65)	56 ± 8 (36–68)	0.45
NYHA Class I	79 (87.8)	40 (88.9)	39 (86.7)	0.75
NYHA Class II	7 (7.8)	2 (4.4)	5 (11.1)	0.24
NYHA Class III	4 (4.4)	3 (6.7)	1 (2.2)	0.31
QRS duration	104 ± 15	105 ± 15	102 ± 15	0.26
QTc duration	412 ± 23	412 ± 24	411 ± 22	0.80
CTO characteristics*				
Patients with ≥2 vessels with CTO	7 (7.8)	3 (6.7)	4 (8.9)	0.69
Patients with IRA-CTO	24 (26.7)	8 (17.8)	16 (35.6)	0.06
Localization of CTO				
LAD	15 (16.7)	13 (28.9)	2 (4.4)	0.002
RCA	65 (72.2)	28 (62.2)	37 (82.2)	0.03
LCX	17 (18.9)	7 (15.6)	10 (22.2)	0.42
J-CTO registry score ≥2	60/97 (61.8)	28/49 (57.1)	32/48 (66.7)	0.33
Rentrop grade ≤1	26/97 (26.8)	11/49 (22.4)	15/48 (31.3)	0.33
Rentrop grade 2	31/97 (31.9)	14/49 (28.6)	17/48 (35.4)	0.47
Rentrop grade 3	40/97 (41.2)	24/49 (48.9)	16/48 (33.3)	0.12
**Cardiac medication**				
ACE-i or ARB	81 (90.0)	39 (86.7)	42 (93.3)	0.29
Amiodaron	1 (1.1)	1 (2.2)	0	0.32
Antiplatelet therapy	87 (96.7)	44 (97.8)	43 (95.6)	0.56
Betablocker	65 (72.2)	33 (73.3)	32 (71.1)	0.81
Diuretics	10 (11.1)	5 (11.1)	5 (11.1)	0.97
Oral anticoagulation	15 (16.7)	8 (17.8)	7 (15.6)	0.78
Sotalol	3 (3.3)	1 (2.2)	2 (4.4)	0.56
Statin	84 (93.3)	42 (93.3)	43 (95.6)	0.63

Data are presented as n (%) or mean ± standard deviation. Abbreviations: ACE-i, angiotensin-converting enzyme inhibitor; AF, atrial fibrillation; ARB, CTO, chronic total occlusion; eGFR, estimated glomerular filtration rate; iCVA, ischemic cerebral vascular attack, ILR, implantable loop recorder; J-CTO, Japanese Multicenter CTO Registry score; LAD, left anterior descending coronary artery; LCX, left circumflex coronary artery; MI, myocardial infarction; NYHA, New York Heart Association; RCA, right coronary artery; TIA, transient ischemic attack. *For patients who were treated with PCI-CTO, these were the CTO characteristics prior to PCI.

In group A (n = 45), the indications for CTO revascularization were progressive angina (n = 33, 73.3 %), ischemia detected on cardiac magnetic resonance (CMR) or nuclear scan (n = 6, 13.3 %), and chronic heart failure (n = 4, 8.9 %). In two patients (4.4 %) who were treated with emergent PCI for myocardial infarction, the operator decided for immediate complete revascularization including CTO-PCI. In group B (n-45), there were eight patients (17.8 %) who had a recent failed CTO-PCI. Poor distal flow (Rentrop grade ≤1) was observed in 15 (31.3 %) of CTOs in group B. Furthermore, IRA-CTO was present in 16 (35.6 %) of patients in group B.

### 3.2 Primary endpoint

**Fig. 2:**
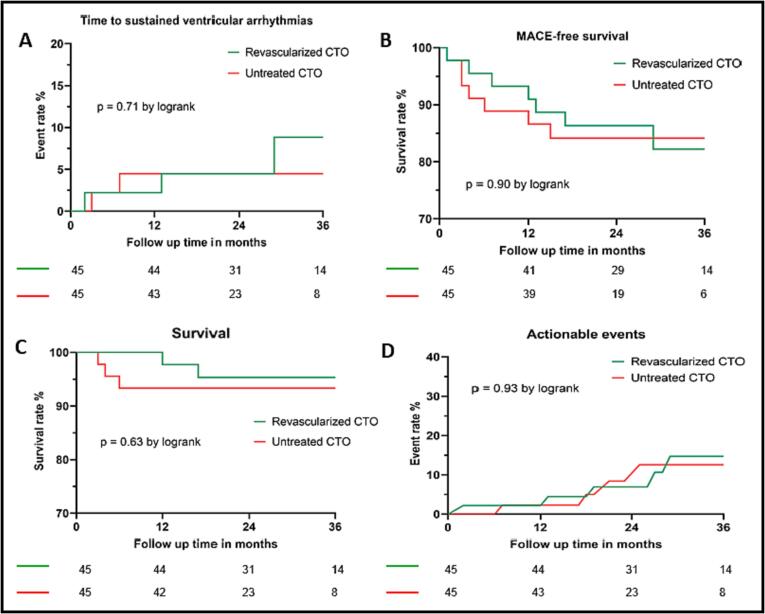
(A) Comparison of the cumulative event rate of sustained VT or VF between patients with revascularized CTO and patients with untreated CTO. (B) Comparison of the cumulative MACE-free survival rate between patients with revascularized CTO and patients with untreated CTO. (C) Comparison of the cumulative survival rate between patients with revascularized CTO and patients with untreated CTO. (D) Comparison of the cumulative event rate of actionable events between patients with revascularized CTO and patients with untreated CTO.

During a median follow-up of 26 months (IQR 19–35), 5 patients (5.6 %) reached the primary endpoint. Their baseline characteristics and detailed primary event characteristics are shown in Table 2. There was no difference in the 3-year cumulative event rate between both groups (8.8 % vs. 4.5 %, P = 0.71, for group A and B, respectively) (Fig. 2A).

**Table 2:** Baseline characteristics and event characteristics of patients with sustained VA.

Patient	Demographics	Relevant medical history	Intervention	Primary event	Outcome
1	59 Y, male	CABG, PCI, LVEF 45 %	Successful PCI IRA-CTO RCA for progressive angina. J-CTO score 3, Rentrop score 2.	Hemodynamic stable sustained monomorphic VT (CL 340 ms, 50 min) after 1 month of follow-up	ICD for secondary prevention, 2 appropriate ICD interventions during follow-up
2	77 Y, male	Chronic AF, hypertension, LVEF 42 %	Succesful PCI CTO LCX and PCI CTO RCA for new-onset heart failure, J-CTO score 3/2, Rentrop score 2/3	Syncope during fast monomorphic VT, (CL 250 ms, 4 min) after 30 months of follow-up	ICD for secondary prevention, revascularization for in-stent restenosis RCA and LCX, recurrent polymorphic VTs, treated with amiodarone.
3	47 Y, male	DM2, hypertension, PCI LCX for NSTEMI, LVEF 53 %	Failed PCI IRA-CTO RCA, J-CTO score 2, Rentrop score 1	OHCA with successful resuscitation because of fast monomorphic VT (CL 200 ms, duration 9 min) after 7 months of follow-up	Successful CTO RCA revascularization, ICD for secondary prevention, multiple appropriate ICD shocks, scheduled for VT ablation
4	77 Y, male	Skin cancer, no history of cardiovascular disease, LVEF 43 %	Medically managed IRA-CTO RCA and CTO LCX, J-CTO score 2/1, Rentrop score 3/2	OHCA because of polymorphic VT (CL 240 ms, duration 16 min), unsuccessful resuscitation	NA
5	66 Y, male	No prior cardiovascular history, high cardiovascular risk profile, LVEF 55 %	Succesful PCI CTO RCA, J-CTO score 2, Rentrop score 2	OHCA because of fast polymorphic VT (CL 200 ms, duration 3 min), successful resuscitation	Succesful PCI RCA for subtotal in-stent restenosis, refused ICD implantation

Abbreviations: AF, atrial fibrillation; CABG, coronary artery bypass graft; CL, cycle length; CTO, chronic total coronary occlusion; DM2, diabetes mellitus type 2; ICD, implantable cardioverter defibrillator; IRA, infarct related artery; J-CTO score, Japanese Multicenter CTO Registry score; LCX, left circumflex coronary artery; LVEF, left ventricular ejection fraction; OHCA, out-of-hospital cardiac arrest; PCI, percutaneous coronary intervention; RCA, right coronary artery; VT, ventricular tachycardia.

**Fig. 3:**
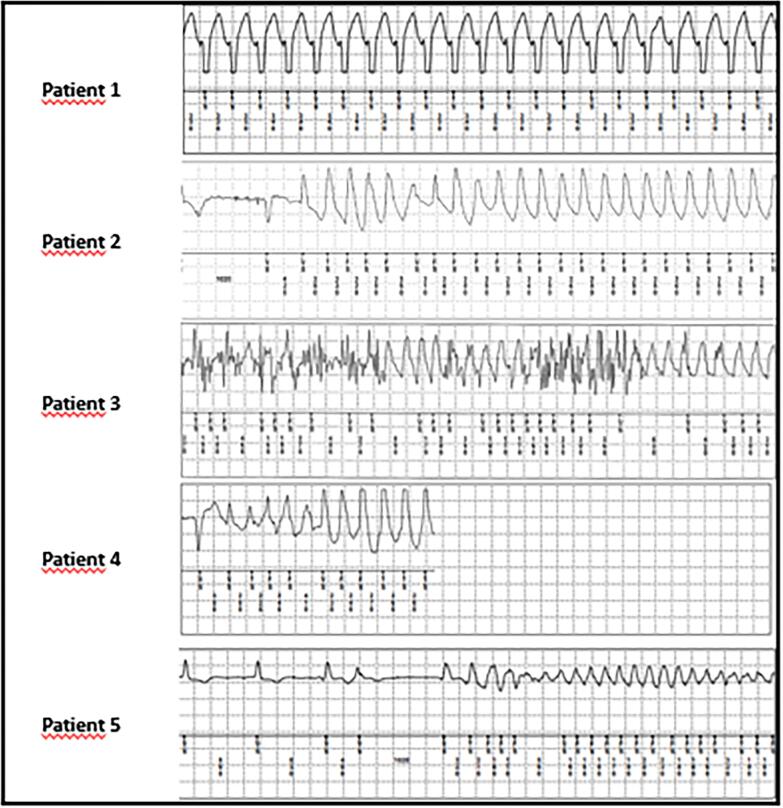
ILR-registration of the primary endpoint per patient.

The first patient was a 59-year-old man with a prior history of coronary artery bypass grafting and PCIs. He was admitted at our hospital because of progressive angina and underwent a successful CTO-PCI of the RCA (group A) with resolution of his symptoms. One month after ILR implantation he experienced chest pain during gardening and he went to bed to rest. ILR recording demonstrated that he had a stable fast monomorphic VT (cycle length (CL) 340 ms) during this episode (Fig. 3, patient 1). The episode lasted 50 min with spontaneous termination. He received an VVI-ICD and had two appropriate ICD interventions during follow-up.

The second patient was a 77-year-old man with a history of permanent atrial fibrillation and hypertension. He underwent coronary angiography for new onset heart failure which demonstrated multivessel CTO (left circumflex [LCX] and RCA). He underwent successful CTO-PCI of both lesions (group A). After a follow-up of 30 months he had a syncopal episode and documentation of a fast monomorphic VT (CL 250 ms) triggered by a short-coupled premature ventricular complex (Fig. 3, patient 2). Coronary angiography demonstrated in-stent restenosis of both stents in the LCX and RCA and he underwent complete percutaneous revascularization. He received an VVI-ICD and had experienced recurrent polymorphic VAs which were treated with amiodarone.

The third patient was a 47-year-old man with a medical history of diabetes mellitus, hypertension, and a previous non-ST-segment elevation myocardial infarction (NSTEMI) treated with PCI LCX and a failed CTO-PCI RCA (group B). He had a preserved LV function. After 7 months of follow-up he experienced an out-of-hospital cardiac arrest and was successfully resuscitated. At the time of cardiac arrest a very fast monomorphic VT (CL 200 ms) was documented by the ILR with a duration of nine minutes (Fig. 3, patient 3). A coronary angiogram did not show new coronary stenosis. He was treated with a CTO-PCI RCA and received a VVI-ICD for secondary prevention. During follow-up he experienced several appropriate ICD shocks due to recurrent fast monomorphic VT.

The fourth patient was a 77-year-old male with a medical history of skin cancer. He was admitted after successful resuscitation from an out-of-hospital cardiac arrest with new-onset left bundle branch block. A coronary angiography showed stenosis of the left main coronary artery and two CTOs (RCA, LCX). The left main was treated successfully with PCI. The patient was transferred for further revalidation to a peripheral hospital. It was decided that the residual CTO lesions would be treated conservatively (group B). No ICD implantation was performed because the VF was considered secondary to extensive cardiac ischemia from the left main coronary stenosis in combination with the multivessel CTO. After 3 months of follow-up he experienced another out-of-hospital cardiac arrest with documentation of a polymorphic VT (CL 240 ms), triggered by a short-coupled premature ventricular complex with degeneration to VF (Fig. 3, patient 4). Resuscitation was not successful.

The fifth patient was a 66-year-old man with a high cardiovascular risk profile who underwent surgical treatment of rectal cancer which has a complicated course. During his stay at the surgical ward, he experienced a NSTEMI for which he underwent a coronary angiography demonstrating multi-vessel coronary disease. A multi-vessel PCI, including a CTO-PCI RCA, was performed successfully. After recovery and hospital discharge, the patient was included in the present study (group A). He had a preserved LV function. After a follow-up of 13 months he experienced an out-of-hospital cardiac arrest with successful resuscitation. The ILR documented a fast polymorphic VT that quickly degenerates in VF (Fig. 3, patient 5). He recalled some mild angina the last couple of months during cycling for which he did not seek medical contact. A coronary angiography demonstrated subtotal in-stent restenosis of the RCA and two intermediate lesions of the left main and left anterior descending artery. Successful multi-vessel PCI was performed. Patient was offered an ICD as secondary prevention but refused because of his work as a truck driver.

### 3.3 MACE and all-cause mortality

MACE occurred in 14 patients (15.6 %): death (n = 5, 5.6 %), urgent revascularization (n = 4, 4.4 %), myocardial infarction (n = 3, 3.3 %), stroke (n = 2, 2.2 %). The 3-year cumulative event rate was similar between groups (17.8 % vs. 15.9 %, P = 0.90, for group A and B, respectively) (Fig. 2B). In total, 5 patients (5.6 %) died during the study period. Most patients died due to a non-cardiovascular cause: COVID-19 infection (n = 2, 2.2 %), complications of abdominal surgery (n = 1, 1.1 %), and esophageal cancer (n = 1, 1.1 %). Only 1 patient (1.1 %) had a sudden cardiac death. The 3-year cumulative survival rate was similar between groups (95.3 % vs. 93.3 %, P = 0.63, for group A and B, respectively) (Fig. 2C).

### 3.4 Actionable arrhythmic event

During follow-up, 11 patients (12.2 %) had an actionable arrhythmic event based on the findings of the ILR. Actionable arrhythmic events were de novo atrial fibrillation (n = 4, 4.4 %), sustained VA (n = 4, 4.4 %), sinus arrest (n = 2, 2.2 %), and nonsustained VT (n = 1, 1.1 %). The 3-year cumulative actionable arrhythmic event rate was similar between groups (14.7 % vs. 12.6 %, P = 0.93, for group A and B, respectively) (Fig. 2D).

## 4 Discussion

We evaluated the incidence of life-threatening VA in patients with treated and untreated CTO who were not a candidate for an ICD. The 3-year cumulative event rate was 8.8 % and 4.5 %, for patients with treated and untreated CTO, respectively. To our knowledge, this is the first prospective multicenter study which has focused specifically on the incidence of VA in CTO patients using continuous arrhythmia monitoring. We did not include patients with severe LV dysfunction who would qualify for an ICD as primary prevention.

### 4.1 Incidence of ventricular arrhythmias in ischemic cardiomyopathy using ILR

Previous studies have evaluated the incidence of arrhythmias using an ILR in patients with ischemic cardiomyopathy. The *Cardiac Arrhythmias and Risk Stratification after Myocardial Infarction* (CARISMA) trial was the first prospective, multicenter study (published in 2010) that investigated the incidence and prognostic significance of ILR-detected arrhythmias in 297 patients with reduced LVEF (≤40 %) after a recent myocardial infarction [13]. During a median follow-up of 23 months, the incidence of sustained VA was **5.7 %** (sustained VT 3.0 %, VF 2.7 %).

Recently, the *Implantable cardiac monitors in high-risk post-infarction patients with cardiac autonomic dysfunction and moderately reduced left ventricular ejection fraction* (SMART-MI) was published [14]. In SMART-MI 400 patients with acute myocardial infarction, autonomic dysfunction, and moderately reduced LVEF (36–50 %) were randomized to an ILR or conventional follow-up. During a median follow-up of 21 months, the incidence of sustained VA was **3.0 %** in the ILR group and 1.0 % in the conventional non-ILR group (P = 0.19). Continuous arrhythmia monitoring increases the detection of VA. One of our study patients (patient 1, Table 2) only experienced chest pain during sustained VA. Without an ILR, the sustained VA episode would have been missed.

The incidence of sustained VA using ILR in our study (5.6 % during a median follow-up of 26 months) is comparable to the incidence of sustained VA in the CARISMA trial, but higher than the ILR-arm of the SMART-MI trial. Interestingly, the CARISMA trial only included patients with LVEF ≤40 %, while we included patients with moderately reduced or preserved LVEF (>35 %). Abovementioned observations may suggest that a CTO is a risk modifier for the occurrence of VA which seems to be stronger than cardiac autonomic dysfunction.

### 4.2 Effect of CTO revascularization on ventricular arrhythmic risk

Ventricular arrhythmogenesis in CTO patients has been postulated to be multifactorial: the presence of a myocardial scar with an ischemic border zone, chronic residual ischemia despite collaterals, heterogeneity in repolarization, and hibernating myocardium may favor VT [15]. CTO revascularization relieves ischemia in the territory of the CTO by restoring coronary flow. Literature on the effect of CTO-PCI on the incidence of VA is sparse and this limits the risk–benefit analysis of performing a time-consuming, expensive, complex revascularization procedure for this specific indication (i.e., reduction of VA burden). Small paired electrophysiological studies with 3D mapping of the LV have shown reverse electrical remodeling with reduction of ischemic scar border zone after successful CTO-PCI [10], [11]. A smaller border zone may be associated with a lower arrhythmic risk. A large Italian multicenter observational study by Godino et al. (baseline LVEF 53 ± 10 %) demonstrated that a failed CTO-PCI was associated with an almost threefold increased risk of sudden cardiac death and/or sustained VA even after correcting for confounders such as LVEF [16]. But a failed CTO-PCI is not similar to a conservatively treated CTO. Furthermore, large Italian observational studies showed that among patients with ischemic cardiomyopathy and an ICD (i.e., reduced LVEF), successful CTO revascularization, especially successful IRA-CTO-PCI, was associated with a lower rate of appropriate ICD therapy in comparison to CTO patients who were treated medically [17], [18].

In contrast, a study from Leipzig demonstrated that in patients with ischemic cardiomyopathy who underwent VT ablation, successfully revascularized CTO patients had a two-fold higher risk of VT recurrence in comparison to patients with untreated CTO [19]. Inhomogeneity of sympathetic innervation in patients with hibernating myocardium that persists after revascularization may provide a partial explanation for this phenomenon [20], [21]. Interestingly, there were two patients with successful CTO-PCI in our study population who experienced a sustained VA during follow-up and were found to have an in-stent restenosis. The in-stent restenosis is the most likely cause of the occurrence of VA and might conceal a possible benefit of CTO revascularization.

Furthermore, we observed that in patients with successful CTO revascularization the morphology of the sustained VT was monomorphic in the majority of cases (2 out of 3). In contrast, the VT was polymorphic in the only patient with untreated CTO with sustained VT. This may be related to relief of cardiac ischemia in the successful CTO revascularization group reducing the risk of polymorphic VT. It is obvious that a prospective randomized trial with large sample size is needed to provide definite answers on the benefit of CTO-PCI regarding the risk of VA.

Currently, the *Nordic Baltic CTO arrhythmia study* (CTO-ARRHYTHMIA, NCT04542460) trial is recruiting patients (target 200 patients). This is a multicenter randomized trial evaluating the impact of CTO-PCI on ILR-detected clinically significant arrhythmias in comparison to conservative treatment [22]. The first results are expected mid-2024 and will hopefully shed more light on the benefit of CTO-PCI on arrhythmic risk.

## 5 Limitations

This study has several limitations. First, this was a nonrandomized trial, thus, conclusions regarding differences between groups should be drawn with caution considering the inherent selection bias. Second, the sample size was small, especially the number of patients with a failed CTO-PCI was low (9 %), therefore, no specific conclusions can be made regarding this group. This group is of particular interest because prior studies have shown an increased arrhythmic risk after a failed CTO-PCI.(16) This low number of patients with failed CTO-PCI reflects the high procedural success due to dedicated CTO operators and improved CTO devices. Third, within the PCI-CTO group, a higher prevalence of patients experiencing angina was observed, with a more frequent occurrence of a CTO in the LAD. Fourth, the ILR algorithm for tachycardia detection was set at 176 beats per minute, therefore slow VA might have been missed by the ILR. Finally, considering the relationship between the presence of myocardial scar and VA, it would be informative if we had systematically evaluated the presence of myocardial scar with cardiac magnetic resonance imaging in our study population. This should be the goal of future studies.

## 6 Conclusion

Patients with a CTO, who do not qualify for an ICD, have a substantial risk of sustained VA. CTO may be an important risk modifier for the occurrence of VA in patients with moderately reduced and preserved LVEF. Randomized trials are necessary to evaluate the antiarrhythmic effects of a CTO-PCI.

## CRediT authorship contribution statement

**Amira Assaf:** . **Rafi Sakhi:** Funding acquisition, Investigation, Methodology, Project administration, Writing – review & editing. **Roberto Diletti:** Conceptualization, Supervision, Writing – review & editing. **Alexander Hirsch:** Conceptualization, Resources, Supervision, Writing – review & editing. **Cornelis P. Allaart:** Investigation, Resources, Supervision, Writing – review & editing. **Rohit Bhagwandien:** Writing – review & editing. **Mehran Firouzi:** Resources, Writing – review & editing. **Pieter C. Smits:** Resources, Writing – review & editing. **Mark G. Hoogendijk:** Supervision, Writing – review & editing. **Dominic A.M.J. Theuns:** Conceptualization, Supervision, Writing – review & editing. **Sing-Chien Yap:** Conceptualization, Funding acquisition, Methodology, Project administration, Resources, Supervision, Validation, Visualization, Writing – review & editing.

## Declaration of competing interest

The authors declare the following financial interests/personal relationships which may be considered as potential competing interests: SCY has received consultancy and speaker fees from Boston Scientific, and institutional research grants from Medtronic, Biotronik and Boston Scientific. The other authors have nothing to declare.
